# Experimental and Theoretical Study of Sorption Capacity of Hexagonal Boron Nitride Nanoparticles: Implication for Wastewater Purification from Antibiotics

**DOI:** 10.3390/nano12183157

**Published:** 2022-09-12

**Authors:** Liubov Yu. Antipina, Kristina Yu. Kotyakova, Mariya V. Tregubenko, Dmitry V. Shtansky

**Affiliations:** Laboratory of Inorganic Nanomaterials, National University of Science and Technology “MISIS”, 4 Bld.1 Leninsky Av., Moscow 119049, Russia

**Keywords:** BN nanoparticles, sorption capacity, antibiotics, wastewater purification, DFT calculations

## Abstract

The constant accumulation of antibiotics and their degradation products in wastewater as a result of human activity poses a serious threat to humanity and other living beings. To contribute to solving this important problem, hollow hexagonal boron nitride nanoparticles (BNNPs) with a spherical shape and smooth surface were synthesized, which were characterized as an efficient adsorbent for wastewater treatment from three types of antibiotics: ciprofloxacin (CIP), tetracycline (TC), and benzylpenicillin (BP). As follows from DFT calculations, the interaction of antibiotic molecules (AM) with the BN surface is neither purely physical nor purely chemical, and negative binding energy (*BE*) indicates that the adsorption process is spontaneous and endothermic. The calculated electron density redistributions at the AM/BN interfaces show that antibiotics interact with BN mainly through oxygen-containing groups. In addition, this interaction causes the BN surface to bend, which increases both the *BE* and the contact area. The removal efficiency of antibiotics (*Re*, %) depends on their initial concentration. At an initial concentration of 10 µg/mL, *Re*_50_ and *Re*_100_ were observed after 24 h and 14 days, respectively. With an increase in the initial concentration to 40 μg/mL, *Re*_50_ and *Re*_100_ were achieved after 5 and 28 days (with the exception of ciprofloxacin (~80% *Re*)). The maximum sorption capacity of BNNPs (*q_e_*) was determined to be 297.3 mg/g (TC), 254.8 mg/g (BP), and 238.2 mg/g (CIP), which is significantly superior to many other systems. Tetracycline is adsorbed much faster than the other two antibiotics, which is confirmed by both theoretical and experimental data. Based on the results of the DFT analysis, a simple and efficient sorbent regeneration strategy was proposed, which ensures complete removal of antibiotics after 14 (BP), 21 (TC), and 10 (CIP) days. Thus, the obtained results clearly show that BNNPs are promising sorbents for various classes of antibiotics, including aminoglycosides, tetracyclines, and β-lactams.

## 1. Introduction

The uncontrolled, ever-growing accumulation of antibiotics and their residues in the environment is an acute modern problem. Their presence in water and soil is a potential hazard to the environment, humans, and other living beings. Many therapeutic agents are not completely metabolized, which leads to the penetration of active drug molecules into the biological environment, the emergence of new contamination sources, and the wide spread of bacteria and microorganisms with multidrug resistance [1,2,3]. Modern wastewater facilities do not allow efficient removal of antibiotic residues from the environment [4,5], which leads to their accumulation in ecological systems [6,7]. Global studies of river pollution with antibiotics have shown that 65% of surveyed rivers in 72 countries on 6 continents are contaminated with antibiotics [8]. In some rivers, the concentrations were so high that they posed a real danger to both the ecosystem and human health. Therefore, the development of effective approaches to the removal of antibiotics from the aquatic environment is of great importance.

The removal of antibiotics and their residues from water and wastewater prior to their final release into the environment is of particular concern [9]. Modern purification methods can be roughly divided into the following three categories depending on the purification mechanism: biological treatment [10,11], chemical degradation [9,12], and physical removal. Each of these methods has its own advantages and disadvantages. For example, biological purification can remove most antibiotic residues, but the introduction of active organisms into the aquatic environment can upset the ecological balance. Various chemical approaches (ozonation, chlorination, and Fenton oxidation) cannot provide complete purification and, in some cases, lead to the death of beneficial microorganisms due to low selectivity. Photocatalysis is widely used in new environmental control strategies [13,14,15]. However, this method has a number of key disadvantages, such as insufficient use of visible light, rapid annihilation of photogenerated carriers, and incomplete mineralization, which greatly limits its application [9]. The adsorption of drug molecules on a safe carrier is the most preferred purification method since this approach allows the use of simple chemical-physical processes to purify water, air, and surfaces from organic contaminants, which makes this process environmentally friendly and economical. A good alternative is to combine physical antibiotic removal methods with chemical degradation. This combination can significantly reduce wastewater toxicity by removing antibiotic residues.

Nanomaterials with a high specific surface area are a promising platform for the development and production of low-cost and highly efficient sorbents for various pollution molecules [16,17]. For example, graphene-based nanomaterials were utilized to remove antibiotics [18,19,20], which are adsorbed on the material surfaces due to π-π-, electrostatic or hydrophobic interactions, as well as the formation of hydrogen bonds. Highly efficient antibiotic sorption was also observed when using highly porous, surface-active, and structurally stable silica-based materials [21,22], metal oxide nanoparticles [15,23,24], and metal-organic frameworks [25,26].

Due to the unique combination of physicochemical properties, hexagonal boron nitride (h-BN) finds application in the following various fields: physics, chemistry, materials science, and biomedicine [27,28,29]. Its high specific surface area and superior thermal and chemical stability determine its attractiveness as an effective sorbent. The polarity of the BN bonds and the large surface area provide good adsorption properties for various substances, from organic pollutants [30] to hydrogen [31]. Since BN nanostructures are very light, BN-based sorbents have a high gravimetric capacity, and their high chemical and thermal stability ensure good material regeneration. Hexagonal BN mesoporous fibers [32] and h-BN porous whiskers [33] showed a high degree of sorption of organic colorants (up to 631 mg/g). Cotton flower-like porous BN [34] and stamen-shaped porous boron carbon nitride nanoscrolls [35] also demonstrated highly efficient removal of contaminants. Hollow BN nanoparticles can serve as a reservoir of boron for the treatment of prostate cancer [36].

The aim of this work is to study the removal efficiency of various classes of antibiotics (aminoglycosides (ciprofloxacin), tetracyclines (tetracycline), and β-lactams (benzylpenicillin)) present in high concentrations in wastewater [37], using BN nanoparticles (BNNPs). For each class of antibiotics, the maximum adsorption capacity of BNNPs was determined. The mechanisms of antibiotic adsorption and subsequent sorbent purification were analyzed based on quantum chemical modeling, and the binding energies and electron density redistributions were determined. A simple and efficient sorbent regeneration strategy has also been proposed.

## 2. Materials and Methods

### 2.1. Preparation of BN Nanoparticles

BNNPs were synthesized by chemical vapor deposition (CVD) in an induction-heated vertical reactor by reacting ammonia with boron oxide vapor (BO, B_2_O_2_) resulting from the thermal dissociation of a boron oxide precursor, as described elsewhere [38].

### 2.2. Structural Characterization of BNNPs

The BNNP morphology was analyzed by scanning electron microscopy (SEM) on a JSM-7600F (JEOL, Tokyo, Japan) instrument equipped with an energy dispersive X-ray (EDX) detector (Oxford Instruments, High Wycombe, UK). The microstructure and phase composition of BNNPs were studied using an FEI Tecnai G2 Spirit Twin transmission electron microscope (Thermo Fisher Scientific, New York, NY, USA) operated at an accelerated voltage of 120 kV and an Ultima IV X-ray diffractometer (Rigaku, Wilmington, MA, USA). Chemical bonds after the antibiotic absorption on the BN surface were determined by Fourier-transformed infrared (FTIR) spectroscopy in the total reflection mode in the range of 400–4000 cm^−1^ with a resolution of 4 cm^−1^. The specific surface area of the samples was measured by low-temperature nitrogen adsorption at 77 K on a Nova 1200e instrument (Quantachrome, Boynton Beach, FL, USA). Before measurements, the samples were degassed at 150 °C. The specific surface area was calculated using the Brunauer–Emmett–Teller (BET) theory.

### 2.3. UV-Vis-Spectrophotometry

Antibiotic concentrations were measured on a Flame UV-Vis spectrophotometer (Ocean Optics, Orlando, FL, USA) in the wavelength range from 190 to 500 nm. A calibration curve was plotted at different antibiotic concentrations (from 0.5 to 4000 µg/mL). The antibiotic concentration in the BNNP solution at each time point was compared with the calibration curve. The optical density (λ max, nm) was also taken into account at an antibiotic concentration of 1 mg/mL. Drug release experiments were carried out in triplicate for each type of antibiotic [39,40,41,42,43].

### 2.4. Adsorption Studies

Adsorption studies were performed using benzylpenicillin (BP), tetracycline (TC), and ciprofloxacin (CIP). Antibiotic solutions were obtained by dissolving tablets of the following compositions:

- Tetracycline: 1 tablet contains the active substance 100 mg of tetracycline hydrochloride;
- Bicillin: powder for suspension for intramuscular injection contains benzathine benzylpenicillin (80%) and benzylpenicillin procaine (20%);
- Ciprofloxacin: 1 tablet contains ciprofloxacin monohydrate (72%), microcrystalline cellulose, corn starch, povidone-CZO, magnesium stearate, polyvinyl alcohol, titanium dioxide, macrogol, and talc.

Each type of antibiotic was completely dissolved in deionized water to prepare a stock solution, which was further diluted to obtain working solutions at antibiotic concentrations of 10, 20, and 40 μg/mL. Then, 50 mg of BNNPs were added to 10 mL of each antibiotic solution and immediately subjected to sonication to obtain a homogeneous suspension. Adsorption tests were carried out at room temperature. Blank experiments were also carried out in the absence of adsorbents to ensure that there were no impurities from the walls of the glass flask.

### 2.5. BNNP Purification from Adsorbed Antibiotics

A mixture of acetonitrile and isopropanol has been shown to be effective in purifying NPs from organic pollutants [44]. In this work, we have developed and studied the following three different methods for purifying BNNPs from antibiotics:

Method 1: Purification in acetonitrile solution (1:6 by volume), acetate buffer solution pH 4.4 (1:6 by volume) and ethanol (4:6 by volume);
Method 2: Purification in pH 4.4 acetate buffer solution;
Method 3: Purification in ethanol solution (1:2 by volume) and acetonitrile (1:2 by volume).

Purification was carried out for 28 days. The concentrations of antibiotics in solution on days 1, 3, 5, 7, 9, 11, 14, 21, and 28 were determined using a UV spectrophotometer. The obtained spectra were integrated and compared with the concentration curve. The experiments were carried out in triplicate, and the obtained data were averaged.

### 2.6. Sorption Kinetic Analysis

To plot adsorption kinetic curves, 2 mL of the supernatant was taken at certain time intervals (8 h, 1, 3, 5, 7, 11, 14, 21, and 28 days) and then the antibiotic concentrations were determined by measuring absorbance values of the solutions using UV-visible spectrophotometry.

The antibiotic removal efficiency (*Re*, %) was calculated using the following equation:

(1) $$Re\left(\%\right)=\frac{\left(C_{0}-C_{t}\right)\times 100}{C_{0}},$$

where *C*_0_ and *C_t_* are antibiotic concentrations at the initial and current (t) time points, respectively (mg/L). The adsorption capacity (*q_e_*, mg/g) of the BNNPs was determined after they were kept in an antibiotic solution at a concentration of 1 mg/mL for 56 days. Value *q_e_* was calculated according to the following equation [45]:

(2) $$q_{e}=\frac{\left(C_{0}-C_{e}\right)\times V}{W},$$

where *C*_0_ and *C_e_* are initial and equilibrium antibiotic concentrations, respectively (mg/L); V—volume of antibiotic solution (L); W—amount of adsorbent (g). Although the antibiotic concentration in wastewater is quite low, in this work we considered concentrations of 10, 20, and 40 µg/mL. All experiments were performed in triplicate.

### 2.7. DFT Calculations of Antibiotic Adsorption on BN Surface

Theoretical analysis of atomic structure and stability of the antibiotic-BN nanosystems was performed using density functional theory (DFT) [46,47] within the generalized gradient approximation (GGA) using normalized Trullier–Martins [48] pseudopotentials in the SIESTA software package [49]. The systems were modeled as supercells with a sufficiently large vacuum gap (at least 20 Å) to neglect intermolecular interactions in the non-periodic direction. The plane wave energy cutoff was set to 200 Ry. To calculate the equilibrium atomic structures, the Brillouin zone was chosen according to the Monkhorst-Pack scheme [50] and selected according to the size of the unit cell from 8 × 8 × 1 to 2 × 2 × 1. To calculate the electronic properties, the cutoff of k-grid was 16 × 16 × 1.

Although the DFT method is widely used to calculate electronic structure, it poorly describes the strength of dispersion and the van der Waals interactions, which can regulate the physical absorption process. Therefore, for modeling antibiotic-BN systems, the Grimme correction method (DFT-D method) [51] was used.

## 3. Results and Discussion

### 3.1. Characterization of BN Nanoparticles

**Figure 1:**
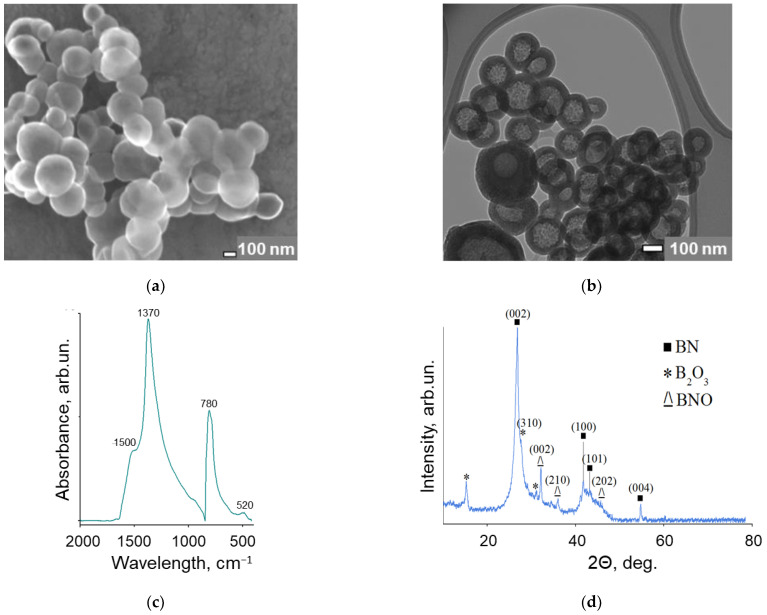
SEM (**a**) and TEM (**b**) micrographs, FTIR spectrum (**c**), and XRD pattern (**d**) of BN nanoparticles.

SEM and TEM micrographs of the as-synthesized BNNPs are shown in Figure 1a,b. Hollow BNNPs has a spherical shape with a thin shell (shell thickness of 30–60 nm) and a smooth surface. The size of BNNPs ranges from 100 to 400 nm, while most NPs (>70%) are from 200 to 300 nm in size.

Figure 1c shows the FTIR spectrum of BNNPs. Two high-intensity peaks can be attributed to out-of-plane B–N–B bending (780 cm^−1^) and in-plane B–N stretching (1370 cm^−1^) vibrations [52]. A small peak at 520 cm^−1^ and a shoulder at 1500 cm^−1^ correspond to the B_2_O_3_ and O-B-O bonds [53]. The XRD pattern of BNNPs is presented in Figure 1d. In addition to the main peaks from the (002), (100), (101), and (004) crystallographic h-BN planes (ICDD card No. 00-034-0421), there are additional maxima corresponding to BNO (ICDD card No. 00-37-1234) and B_2_O_3_ (ICDD card No. 00-06-0297) phases. Thus, the oxidized state of BNNPs observed in the XRD pattern (Figure 1d) is in good agreement with the FTIR spectroscopy data (Figure 1c).

The specific surface area was calculated using the BET model. The specific surface area of nanoparticles with several BN layers, determined by the N_2_ adsorption method, was 33.6 m^2^/g.

### 3.2. Interaction Mechanisms of Antibiotic Molecules with BN Surface: Theoretical Insight

**Figure 2:**
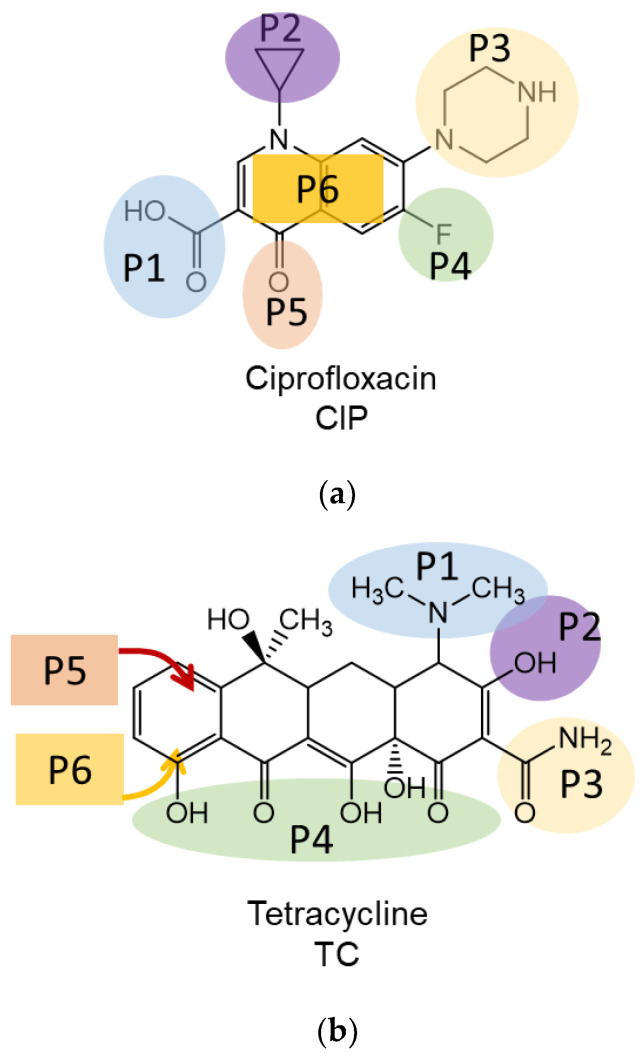

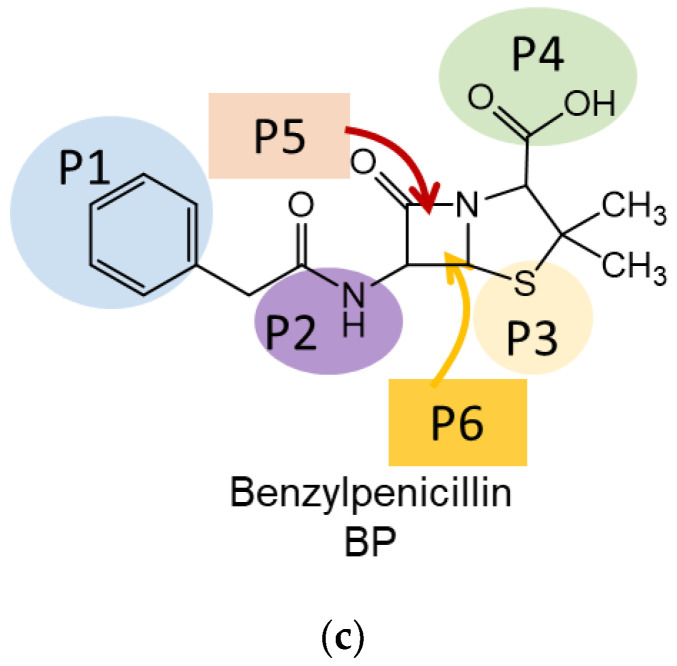
Structures of ciprofloxacin (**a**), tetracycline (**b**), benzylpenicillin (**c**), and their functional groups considered in the theoretical calculations.

The optimal positions of antibiotics relative to the BN surface were theoretically studied. Due to the large size of BNNPs, BN was considered an infinite sheet. First, the preferred positions of antibiotics on the BN surface were determined. For this, six positions were considered for each antibiotic type, corresponding to the main functional groups of antibiotic molecules (Figure 2, Table 1).

As shown in Figure 2a, ciprofloxacin (CIP) contains the following configuration: carboxylic group (P1), cyclopropyl group (P2), pipyrazine ring (P3), fluorine group (P4), oxo group (P5), and a parallel arrangement of the dihydroquinoline ring-vertical stacking (P6). In tetracycline, dimethylamino group (P1), a hydroxyl group, including the simultaneous interaction of both amino groups (P2), amide group (P3), the peripheral region of anthracycline (P4), as well as parallel arrangement with OH groups oriented from (P5) or to (P6) BN plane were considered (Figure 2b). In the case of benzylpenicillin, the following positions were identified: phenylacetyl group (P1), amino group (P2), thioether group (P3), carboxylic group (P4), and two parallel arrangements with the S group oriented from (P5) or to (P6) BN plane (Figure 2c).

For each position, the binding energy (*BE*) was calculated as follows:

(3) $$BE=E_{tot}-E_{\mathrm{BNNPs}}-E_{\mathrm{AB}},$$

where *E_tot_* is the total energy of the system, *E*_BNNPs_ and *E*_AB_ are the energies of a freestanding substrate and an antibiotic molecule, respectively. The *BE* values for antibiotics with different charges on the h-BN surface are present in Table 2. Negatively charged forms of antibiotics (absence of a proton on the carboxylic group) are marked as “−1”, positively charged forms (an additional proton on the amino groups) are denoted as “+1” and uncharged forms of antibiotics are marked as 0.

The most stable position for all antibiotic types is a vertical arrangement, regardless of the molecule charge. In the case of tetracycline, the vertical stacking positions P5 and P6 show rather similar *BE*s (difference up to 1 eV). For benzopenicillin, the most favorable structure is P5 (the thio group is oriented from the BN plane) for the negatively charged form and P6 (the thio group is oriented to the BN plane) for the neutral form of benzopenicillin. Structures P2 and P3 also have rather low *BE*s. It should be noted that the structure of the positively charged benzylpenicillin was not considered in the simulation since it is unstable and is destroyed during optimization. All negatively charged forms of the considered antibiotics (absence of a proton in the -COO- groups) bind more strongly to the surface of BN than neutral or positively charged forms.

For all considered antibiotics, their *BE* with BN is negative and adsorption clusters are more stable than freestanding structures; therefore, adsorption on BN is an exothermic and spontaneous process. The distance between the antibiotic (AB) molecule and the BN surface is more than 2.4 Å.

However, the *BE* is too high for normal physical interaction. To determine the type of interaction in the BN@AB hybrid for each antibiotic type, the electron density redistribution between the entire system ($\rho_{\mathrm{BNNPs}@\mathrm{AB}}^{total}$) and each individual part ($\rho_{\mathrm{BN}}$ and $\rho_{\mathrm{AB}}$ for freestanding h-BN and antibiotic molecule, respectively) was calculated as follows:

(4) $$\rho_{\mathrm{BNNPs}@\mathrm{AB}}^{dis}=\rho_{\mathrm{BNNPs}@\mathrm{AB}}^{total}-\rho_{\mathrm{BN}}-\rho_{\mathrm{AB}}.$$

**Figure 3:**
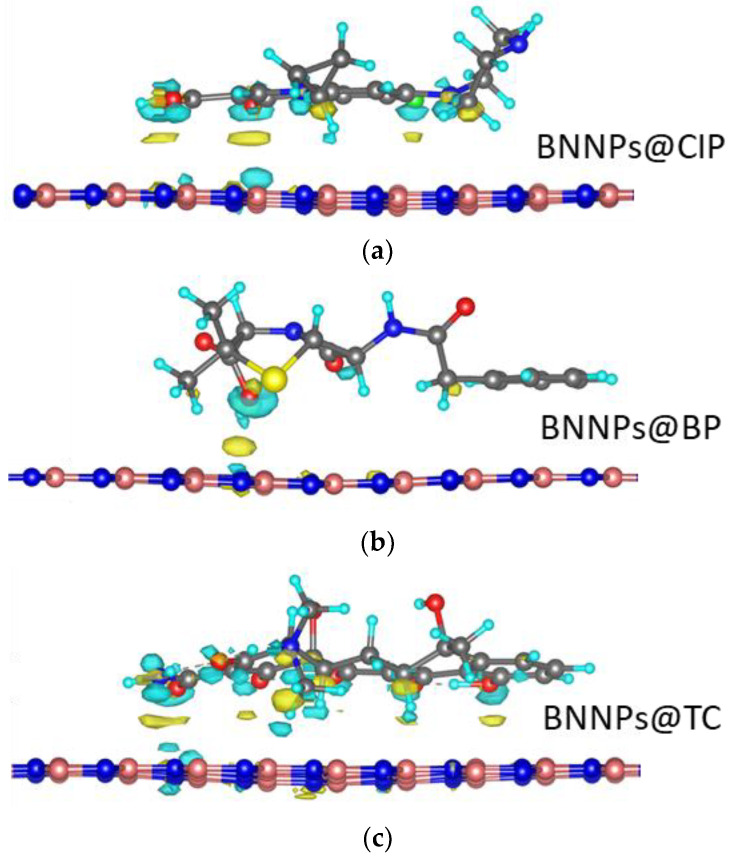
Electron density redistribution at the BN/AB interface. The loss and gain of charge are denoted by yellowish and bluish colors, respectively. The boron, nitrogen, carbon, oxygen, and hydrogen atoms are marked by green, gray, brawn, red, and beige colors, respectively. The isosurface constant value is 0.01 eV/Å.

The structures of BN@AB after geometry optimization and redistribution of electron density near the BN surface for the neutral form of antibiotics are presented in Figure 3.

The obtained results show that the interaction of AB molecules with the BN surface is neither purely physical nor purely chemical. This interaction changes the geometric parameters of the BN surface (Figure 3). As a result of the interaction of AB molecules with functional groups on the BN surface, the electron density is redistributed, which causes the BN surface to bend. The formation of a concave on the BN surface leads to an increase in both the contact area and the *BE*.

The electron density redistribution shows that antibiotics interact with BN mainly through oxygen-containing groups. In the area of -COOH, hydroxyl, and oxo groups, the charge density is transferred to the BN surface, while for other groups (fluorine for CIP, sulfur for BP, and nitrogen-containing groups), the charge is redistributed only on the AB molecule. This explains the higher *BE* of tetracycline compared to benzylpenicillin and ciprofloxacin, which contain a large number of oxygen-containing groups.

### 3.3. Kinetics of Antibiotic Adsorption on BNNPs

**Figure 4:**
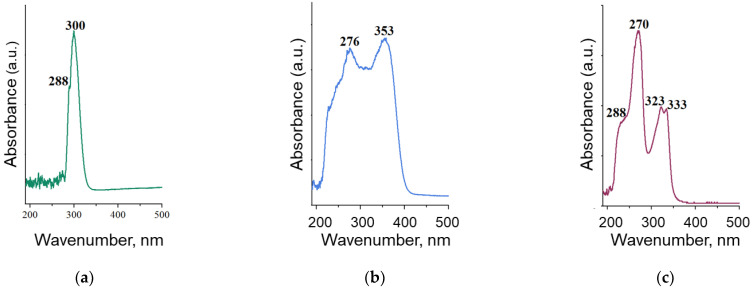
UV-Vis absorption spectra of benzylpenicillin (**a**), tetracycline (**b**), and ciprofloxacin (**c**).

First, the absorption spectra of antibiotics were obtained at a maximum concentration of 200 μg/mL in the wavelength range from 190 to 500 nm using UV-visible spectrophotometry (Figure 4). Absorption peaks were observed at wavelengths of 288 and 300 nm (benzylpenicillin), 276 and 353 nm (tetracycline), and 288, 270, 323, and 333 nm (ciprofloxacin).

**Figure 5:**
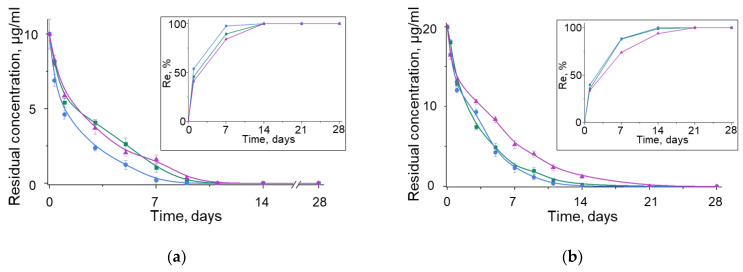

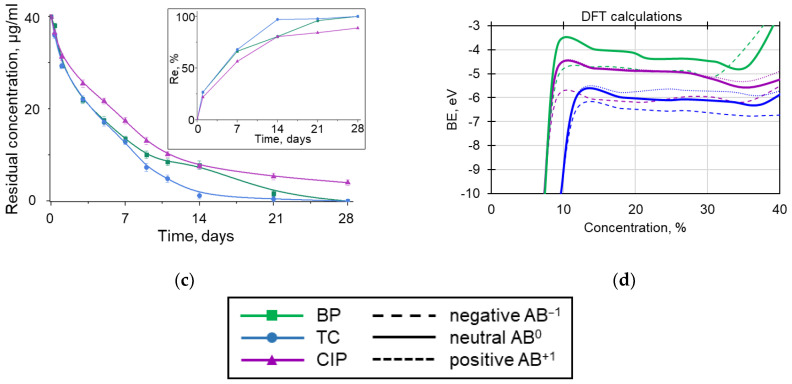
Kinetics of antibiotic adsorption by BNNPs at initial antibiotic concentrations of 10 (**a**), 20 (**b**), and 40 µg/mL (**c**). The studies were carried out at T = 24 °C and pH 6. Insets show the dependence of antibiotic removal efficiency *versus* time. (**d**) DFT calculation of antibiotic sorption. BP—benzylpenicillin, TC—tetracycline, CIP—ciprofloxacin.

The kinetics of change in the antibiotic concentration in the BN suspension was studied for three different initial concentrations (10, 20, and 40 µg/mL) at a temperature of 24°C and pH = 6 for 28 days (Figure 5). The amount of antibiotic adsorbed on the surface of BNNPs per unit time depended on the solution’s initial concentration as follows: the higher the initial antibiotic concentration, the lower the adsorption rate. When testing benzylpenicillin and tetracycline at initial concentrations of 10 and 20 µg/mL, 50% of the antibiotic was adsorbed in the first 2 days. At a concentration of 40 µg/mL, the adsorption time increased to 4 days. In the case of ciprofloxacin, 50% of the antibiotic was adsorbed on days 2, 4, and 6 at concentrations of 10, 20, and 40 µg/mL, respectively. At an initial concentration of 10 μg/mL, 100% adsorption was observed on the 9th (tetracycline) and 11th (benzylpenicillin and ciprofloxacin) days. With an increase in the initial concentration to 20 μg/mL, the time for complete adsorption increased to 14 (tetracycline) and 21 (benzylpenicillin and ciprofloxacin) days. At the maximum studied concentration of 40 µg/mL, only tetracycline and benzylpenicillin showed 100% adsorption on the 28th day, while with the use of ciprofloxacin, the sorption process continued.

The antibiotic removal efficiency (*Re*, %, Equation (1)) was calculated depending on the initial antibiotic concentration (Figure 5a–c, insets). The removal rate in the first days was high in all cases, which can be explained by the abundance of free adsorption sites on the surface of BNNPs that were not occupied by antibiotic molecules. At an initial concentration of 10 µg/mL, a *Re*_50_ was observed in the first 24 h. With an increase in concentration to 20 and 40 μg/mL, the time increased to 3 and 5 days, respectively. It can be seen that, at low initial concentrations (10 and 20 µg/mL), the BNNPs are effective for all three types of antibiotics, and their complete removal from the solution is achieved on days 14 and 21, respectively. At the maximum initial concentration of 40 µg/mL, *Re*_100_ is observed on the 28th day (benzylpenicillin and tetracycline), while ~20% of ciprofloxacin still remains. The *Re* can be arranged in the following order: TC > BP > CIP.

Experimental sorption data were compared with the results of DFT calculations (Figure 5d). For theoretical simulations, the BN cell size with one antibiotic molecule per one h-BN sheet varied from 7 × 13 Å (~43 wt%) to 26 × 27 Å (~9.2 wt%). After reaching a concentration of 13–14 wt% (one antibiotic molecule per 20 × 21 Å BN cell), the *BE* values of all antibiotics decrease. This indicates that the adsorption process is thermodynamically favorable up to sufficiently high antibiotic concentrations. The *BE* values for the BN@TC nanohybrid are lower than those of the BN@CIP and BN@BP counterparts, which is in good agreement with the experimental data that tetracycline is adsorbed much faster than the other two antibiotics. At ~35 wt% and high concentrations (10 × 13 Å and less h-BN cell), the *BE* sharply increases. This is due to the influence of steric factors; antibiotic molecules begin to interact with each other, and the force of their repulsion makes a large contribution to the *BE*.

**Figure 6:**
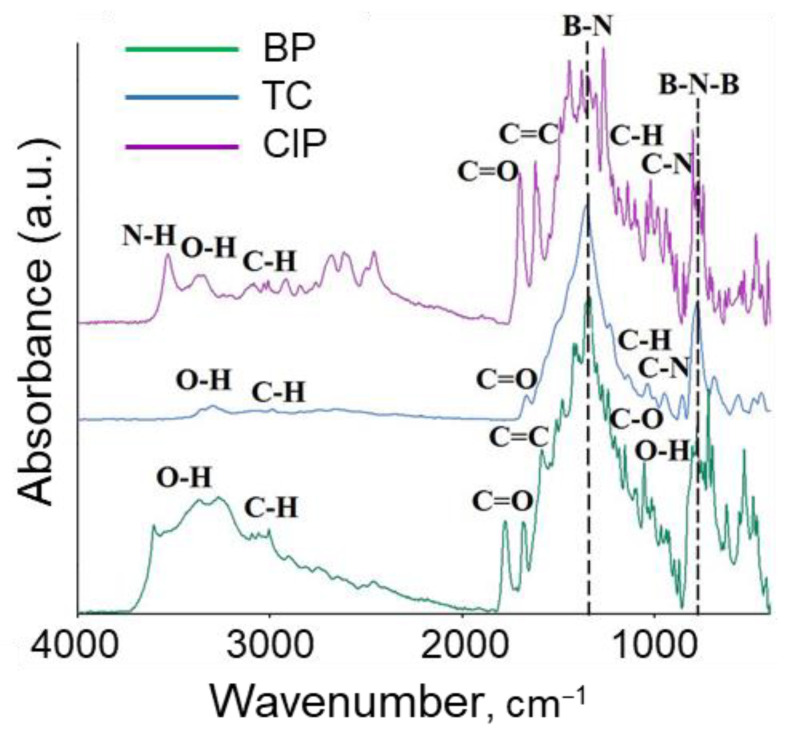
FTIR spectra of BNNPs after adsorption of benzylpenicillin (BP), tetracycline (TC), and ciprofloxacin (CIP).

After completion of the adsorption experiments using the maximum concentration of antibiotics (40 µg/mL), BN@AB samples were analyzed by FTIR spectroscopy (Figure 6). In all cases, high-intensity peaks were present, corresponding to vibrations of bonds in BNNPs (B-N-B at 780 cm^−1^, B-N at 1370 cm^−1^) [52]. Additionally, characteristic peaks of various functional groups present in antibiotics were observed, which confirms their successful adsorption on the BNNP surface. The benzylpenicillin adsorption is confirmed by absorption peaks at 3388 cm^−1^ (O-H stretching vibrations), 3272 cm^−1^ (C-H stretching vibrations), 3004 cm^−1^ (C-H stretching vibrations), 1701 cm^−1^ (C=O group), 1644 cm^−1^ (isolated C=C), 1419 cm^−1^ (C-H stretching vibrations), 1360 cm^−1^ (C-H bending vibrations), 1232 cm^−1^ (C-O stretching vibrations), and 1093 cm^−1^ (O-H bending vibrations) [53]. The BNNPs@TC spectrum contains vibrational peaks characteristic of tetracycline at 3342–3325 cm^−1^, which can be attributed to the stretching vibrations of N-H and O-H bonds. Adsorption bands in the range of 3064–3003 cm^−1^ and 2955–2835 cm^−1^ are ascribed to C-H and CH_3_ stretching vibrations, respectively. The presence of the C=C bond appears as adsorption bands at 1622–1569 cm^−1^. The characteristic peaks at 1454 and 1357 cm^−1^ can be due to the bending vibrations of C-H and CH_3_, respectively. The peaks at 1247–1000 cm^−1^ and 995 cm^−1^ were assigned to the C-H in-plane deformation and the C-N stretching vibration, respectively [54]. The peak at 1270 cm^−1^ observed in the FTIR spectrum of the BNNPs@CIP sample is due to the C-N bond. The remaining adsorption peaks were identified as C=C bond (1680–1620 cm^−1^), C=O bond (1725–1705 cm^−1^), and C-H bond (3150–2750 cm^−1^). A broad maximum in the range 3700–3000 cm^−1^ is associated with the vibrations of N-H and O-H bonds [55].

The maximum sorption capacity (*q_e_*) was determined using Equation (2). After keeping the BNNPs in an antibiotic solution with a concentration of 1 mg/mL for 56 days, the following *q_e_* values were obtained: 297.29 mg/g (TC), 254.76 mg/g (BP), and 238.17 mg/g (CIP). Table 3 compares the values of the maximum adsorption capacity of benzylpenicillin, tetracycline, and ciprofloxacin using different sorbents. It can be seen that BNNPs show a much higher efficiency in removing antibiotic molecules compared to many other materials.

### 3.4. BNNP Purification Strategy from Adsorbed Antibiotics

To be cost-effective, a sorbent used in wastewater treatment must easily regain its adsorption properties after being cleaned of contaminants so that it can be reused. There are chemical, mechanical, dispersive, and mixed methods of cleaning sorbents. Chemical cleaning is based on the use of various chemicals to remove antibiotics. Mixtures of acetonitrile and isopropyl alcohol are highly effective for cleaning contaminated particles [44]. According to the results of our DFT analysis, the *BE* of positively charged forms of antibiotics corresponding to an acidic environment is lower than that of neutral or negatively charged forms (Figure 5d). Thus, three different methods for cleaning BNNPs from antibiotics were developed and tested, as described below.

**Figure 7:**
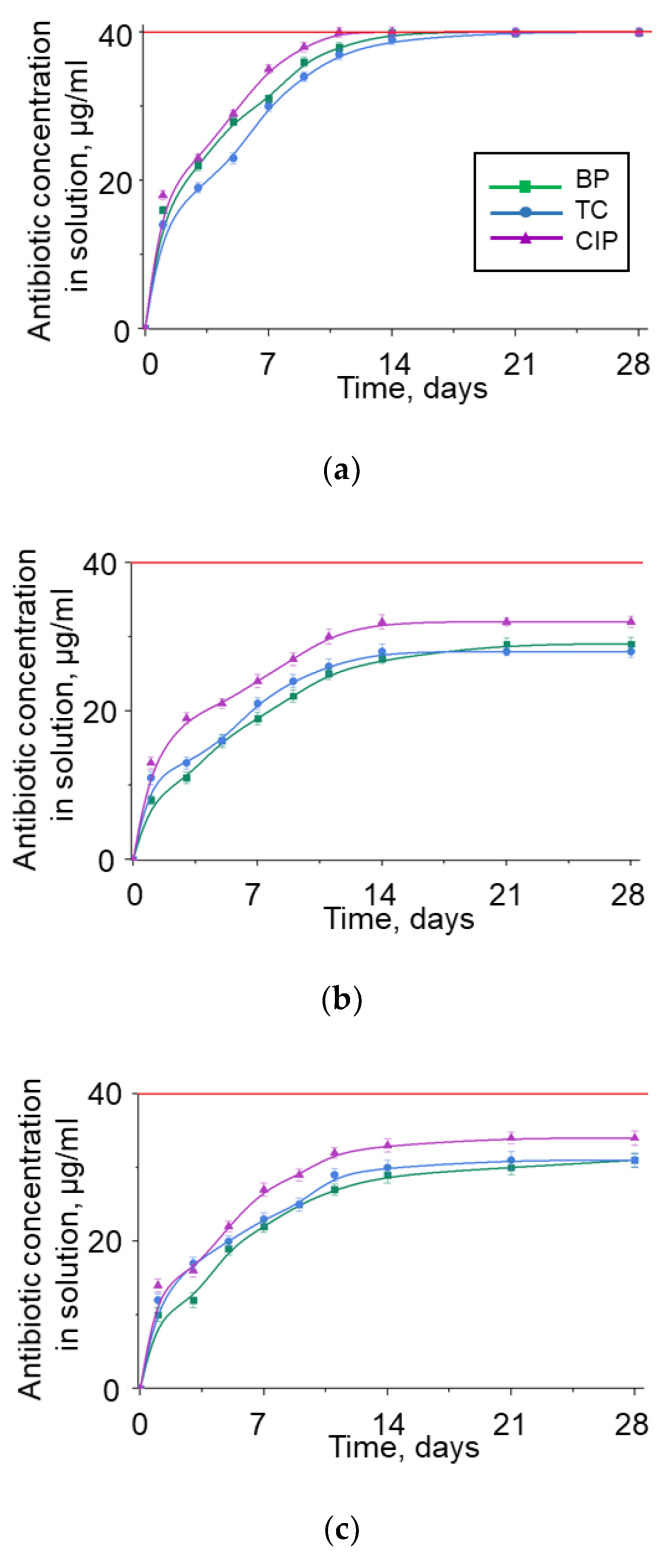
Kinetics of BNNPs purification from antibiotics at an initial antibiotic concentration of 40 μg/mL. Method 1 (**a**); Method 2 (**b**); Method 3 (**c**).

Figure 7 shows the efficiency of BNNP purification from adsorbed antibiotics. Method 1 ensured complete removal of antibiotics after 14 (benzylpenicillin), 21 (tetracycline), and 10 (ciprofloxacin) days. Already on the first day, 40, 35, and 45% of adsorbed benzylpenicillin, tetracycline, and ciprofloxacin, respectively, are released. When using Method 2, the maximum degree of purification from benzylpenicillin, tetracycline, and ciprofloxacin is 72.5, 70, and 80% after 29, 28, and 32 days, respectively, and on the first day, only 20, 27.5, and 32.5% of each antibiotic are desorbed. The efficiency of Method 3 was in the middle between Methods 1 and 2. The degree of BNNP purification from benzylpenicillin, tetracycline, and ciprofloxacin is 25, 30, and 35% (after one day) and 77.5, 77.5, and 85% (after 28 days), respectively.

After cleaning the adsorbents from antibiotics, antibiotic-contaminated solutions can be properly disposed of or recycled using ozonation, chlorination, or Fenton oxidation methods [9,12].

## 4. Conclusions

Here we demonstrate that spherical hollow hexagonal BN nanoparticles (BNNPs) with an average size of 200–300 nm are promising sorbents for wastewater treatment from various types of antibiotics. The maximum sorption capacity of tetracycline, bencylpeniciline, and ciprofloxacin is 297.3, 254.8, and 238.2 mg/g, respectively. The removal efficiency of antibiotics (*Re*, %) depends on their initial concentration; *Re*_50_ and *Re*_100_ are achieved after 24 h and 14 days (10 µg/mL), 3 and 21 days (20 µg/mL) and 5 and 28 days (40 µg/mL), respectively. The mechanisms of antibiotic adsorption and subsequent sorbent purification were analyzed using DFT calculations. The obtained results show that the interaction of antibiotic molecules (AM) with the BN surface is neither purely physical nor purely chemical. Antibiotics interact with BN mainly through oxygen-containing groups, and the adsorption process is spontaneous and endothermic. In addition, this interaction causes the BN surface to bend, which increases both the binding energy and the contact area. Based on the results of the DFT analysis, a simple and efficient BNNP purification strategy was proposed. Combined treatment in a mixture of acetonitrile and ethanol solutions at pH = 4.4 (acetate buffer) ensures complete removal of antibiotics after 14 (BP), 21 (TC), and 10 (CIP) days. Our encouraging results are of great importance for the further development of efficient and cost-effective h-BN-based adsorbents for water purification from therapeutic contaminants.

## Figures and Tables

**Table 1:** Six antibiotic functional groups considered in theoretical calculations.

Position	Ciprofloxacin	Tetracycline	Benzylpenicillin
P1	Carboxylic	Dimethylamino-	Phenylacetyl-
P2	Cyclopropyl-	Hydroxy-	Amino-
P3	Pipyrazine ring	Amide-	Thioether-
P4	Fluoro-	Peripheral region	Carboxylic
P5	Oxo-	Vertical stacking (OH group is oriented from BN plane)	Vertical stacking (thio group is oriented from BN plane)
P6	Vertical stacking of dihydroquinoline ring (vertical stacking)	Vertical stacking (OH group is oriented to BN plane)	Vertical stacking (thio group is oriented to BN plane)

**Table 2:** Binding energies of antibiotics with BN surface.

Position	E_b_, eV							
	CIP			TC			BP	
	−1	0	+1	−1	0	+1	−1	0
P1	−3.66	−1.28	−0.48	−2.91	−2.6	−2.95	−0.07	−0.63
P2	−2.62	−2.38	−3.06	−2.91	−2.59	−2.92	−4.14	−3.12
P3	−1.98	−1.81	−2.46	−2.98	−2.62	−2.01	−4.06	−3.31
P4	−2.58	−2.14	−2.51	−4.26	−4.69	−3.34	−3.37	−2.03
P5	−4.35	−2.07	−1.91	−6.55	−6.36	−6.44	−4.33	−3.14
P6	−5.44	−4.04	−4.78	−6.56	−5.54	−5.93	−4.05	−3.73

**Table 3:** Maximum adsorption capacity of benzylpenicillin (BP), tetracycline (TC), and ciprofloxacin (CIP) when using various adsorbents.

Adsorbent	Sorption Capacity *q_e_*_,_ mg/g	Adsorbent	Sorption Capacity *q_e_*_,_ mg/g	Adsorbent	Sorption Capacity *q_e_*_,_ mg/g
TC		BP		CIP	
Reduced graphene oxide decorated with MnFe_2_O_4_ NPs [20]	41.00	Magnesium oxide nanoparticles [56]	25.66	Nano-sized magnetite [57]	12.73
Pistachio shell coated with ZnO NPs [58]	95.06	Lemna minor [59]	36.18	Magnetic activated carbon/chitosan nanocomposite [60]	90.00
Nano sheet layered double hydroxide Mg/Al [61]	98.04	CoFe_2_O_4_@CuS magnetic nanocomposite [62]	41.00	Magnetic carbon composite, Fe_3_O_4_/C [63]	90.10
Graphene oxide/calcium alginate composite fibers [64]	131.60	Chitosan extracted from Persian gulf shrimp shell [65]	101.44	Activated carbon supported with multivalent carbon nanotubes [66]	150.00
Shrimp shell waste [67]	229.98	Silica NPs [22]	211.35	Ordered mesoporous carbon [68]	233.37
BNNPs *	297.29	BNNPs *	254.76	BNNPs *	238.17

* This study.
